# The impact of hospitalisation to geriatric wards on the use of medications and potentially inappropriate medications - a health register study

**DOI:** 10.1186/s12877-020-01585-w

**Published:** 2020-06-01

**Authors:** Jeanette Schultz Johansen, Kjell H. Halvorsen, Kristian Svendsen, Kjerstin Havnes, Beate H. Garcia

**Affiliations:** 1 Department of Pharmacy, Faculty of Health Sciences, UiT the Arctic University of Norway, N-9037 Tromsø, Norway; 2 Hospital Pharmacy of North Norway Trust, Tromsø, Norway

**Keywords:** Potentially inappropriate medications, Health register data, Drug therapy, EU(7)-PIM list, NORGEP-NH list, Hospitalization, Health services for the aged

## Abstract

**Background:**

The use of potentially inappropriate medications (PIMs) are associated with negative health effects for older adults. The purpose of this study was to apply national register data to investigate the impact of hospitalisation to geriatric wards in Norway on the use of medications and PIMs, and to compare two explicit PIM identification tools.

**Methods:**

We included 715 patients ≥65 years (mean 82.5, SD = 7.8) admitted to Norwegian geriatric wards in 2013 identified from The Norwegian Patient Registry, and collected their medication use from the Norwegian Prescription Database. Medication use before and after hospitalisation was compared and screened for PIMs applying a subset of the European Union (EU)(7)-PIM list and the Norwegian General Practice – Nursing Home (NORGEP-NH) list part A and B.

**Results:**

The mean number of medications increased from 6.5 (SD = 3.5) before to 7.5 (SD = 3.5) (CI:1.2–0.8, *p* < 0.001) after hospitalisation. The proportion of patients with PIMs increased from before to after hospitalisation according to the EU(7)-PIM list (from 62.4 to 69.2%, *p* < 0.001), but not according to The NORGEP-NH list (from 49.9 to 50.6%, *p* = 0.73). The EU(7)-PIM list and the NORGEP-NH list had more than 70% agreement on the classification of patients as PIM users.

**Conclusions:**

Medication use increased after hospitalisation to geriatric wards. We did not find that geriatric hospital care leads to a general improvement in PIM use after hospitalisation. According to a subset of the EU(7)-PIM list, PIM use increased after hospitalisation. This increase was not identified by the NORGEP-NH list part A and B. It is feasible to use health register data to investigate the impact of hospitalisation to geriatric wards on medication use and PIMs.

## Background

The risk of hospitalisations increases with age. In 2018, 25% of the Norwegian population over 70 years had one or more hospitalisations [1]. Large specialised hospitals often have geriatric wards to care for older patients, where one core feature is the presence of a multidisciplinary health care team. For most patients, this team performs a comprehensive geriatric assessment, which also includes reviewing medications [2, 3]. Medication reviews are important as nearly half of hospitalised older adults use potentially inappropriate medications (PIMs) [4]. PIMs are normally defined as medications where the benefits are outweighed by the potential risks of adverse drug events (ADEs). Identification of PIMs is particularly relevant when safer or more effective treatment alternatives exist [5]. In older adults, PIMs are associated with an increased risk of ADEs and hospitalisations and is a public health concern [6].

A medication review may identify and prevent the use of PIMs. Despite this being an integrated part of the geriatric assessment, study results are conflicting concerning the impact of a geriatric ward stay on PIM prevalence [7–9]. Most previous studies have used admission and discharge summaries to determine medication use. We are not aware of studies applying prescribing registries to explore medication and PIM use related to hospitalisations in geriatric wards.

Several tools have been developed to identify PIMs in older adults. These are either explicit (criterion-based) or implicit (judgment-based), or a mix of both. The major advantage of explicit tools are that they are applicable with little clinical judgment, making them ideal for use in registry studies [5].

Due to inter-country variability in medication therapy traditions and the medications available, several country-specific PIM identification tools have been developed [5]. In Norway, two national PIM-lists exist; The Norwegian General Practice (NORGEP) list from 2009 [10], and The Norwegian General Practice Nursing Home (NORGEP—NH) list from 2015 [11]. NORGEP-NH is an updated version of NORGEP, and although developed primarily as a tool for nursing home patients, it can be useful in the general older population and for pharmacoepidemiological research [11]. Recently, The European Union (EU)(7)-PIM list initiative developed an explicit tool to identify and compare PIM use between European countries, including Scandinavian countries [12]. Application of different PIM lists will influence both the type and number of PIMs identified, and it is important to be aware of similarities and differences between the tools and their strength and limitations, both in daily clinical practice and when used in research. No published studies to date have compared PIMs identified applying the EU(7)-PIM list with NORGEP-NH list.

### Aim

The primary aim was to apply national registry data to explore how hospitalisation to a geriatric ward impact use of medication and PIMs use among older adults. The secondary aim was to compare the EU(7)-PIM and the NORGEP-NH list with regards to PIM identification.

## Method

### Study population

**Fig. 1:**
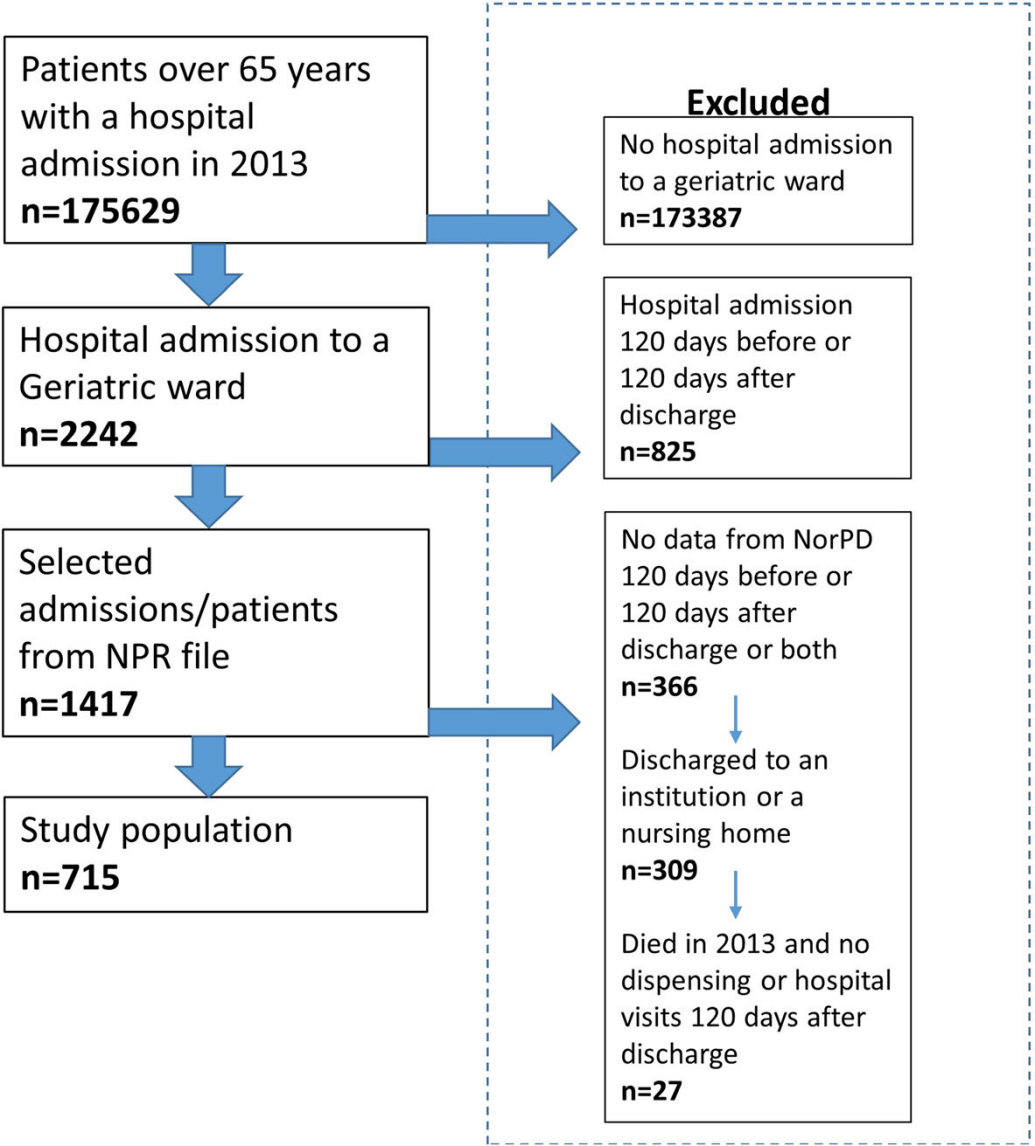
Flowchart of study population selection

We included all patients ≥65 years admitted to geriatric wards in Norway during 2013. We identified patients using data from the Norwegian Patient Registry, holding information on all hospitalisations for all Norwegian citizens through unique personal identification numbers. Their first admission in 2013 was used as their index stay. We excluded all patients with hospital admissions 120 days before or 120 days after discharge from the index hospital stay because we wanted to measure the effect of a single hospitalisation. See Fig. 1 for patient flow.

To identify medication use before and after hospitalisation, we retrieved data from the Norwegian prescription registry, holding information on all dispensed medications from Norwegian pharmacies on an individual level. Because data on medications used during hospital stays, in nursing homes or over the counter medications are not collected by the registry, we excluded patients who were discharged to an institution or nursing home. Patients who died in 2013 were excluded as they could have died in the 120 days following the index stay. If no medication dispensing was identified 120 days before or after discharge from index stay, patients were also excluded (Fig. 1).

### Medication use and comorbidities

We defined medication use before and after hospitalisation as all medications dispensed in the 120 days before and after the index stay, respectively. We chose 120 days because reimbursed medications in Norway (i.e. all medications used for chronic diseases) can only be dispensed for a maximum of 90 days. Consequently, medications dispensed 120 days before and after hospitalisation should represent regular use for chronic conditions, leaving a 30-day window to account for non-adherence and stockpiling. We collected medication data using the medications unique Anatomical Therapeutic Chemical (ATC)-code provided by the World health organisation [13]. We excluded all antibiotics when counting the number of medications (ATC-code: J01), except methenamine, which is commonly used for long term prophylaxis for urinary tract infections.

Information in the Norwegian prescription registry allows for indirect identification of patient comorbidities through reimbursement codes for medications used for chronic diseases. To identify important comorbidities at the time of hospitalisation (description of the study population), we identified reimbursement codes (ICD or ICPC codes) for all medications dispensed 365 days before index hospitalisation and created clinical relevant medical diagnose classes.

### PIM identification

We identified PIM use by applying two explicit tools; the EU(7)-PIM list [12] and the NORGEP-NH list [11]. NORGEP-NH was chosen over NORGEP as it is considered an updated and expanded version of the NORGEP list published in 2009.

From the 282 criteria in the EU(7)-PIM list [12], we applied 263 criteria. We excluded five criteria due to lack of information on the length of therapy (e.g. proton pump inhibitors), 12 criteria specifying medication doses that are unavailable in our dataset and two criteria not specifying ATC codes. See supplement 1 for an overview of exclusions.

From the NORGEP-NH list, we applied all the 26 criteria in part A and B and excluded the de-prescribing criteria in part C as these criteria are most relevant for a nursing home population. We defined “regular use” of hypnotics (criteria 11) as the dispensing of 60 defined daily doses (DDD) or more over 120 days.

### Analysis and statistics

We present continuous variables as means with standard deviation (SD) and categorical variables as proportions. We compared the mean number of medications before and after hospitalisation by applying a dependent paired sample t-test. We compared the proportion of patients with PIM use before or after hospitalisation by applying the related samples McNemar test. Change in the number of identified PIMs before and after hospitalisation was examined applying the related samples Wilcoxon signed-rank test. Agreement in PIM identification between EU(7)-PIM and NORGEP-NH was explored using a Venn diagram. Statistical analysis was performed using IBM SPSS Statistics Version 25.0. A two-sided *P*-value of < 0.05 was considered statistically significant.

## Results

### Study population

Of the 175,629 patients ≥65 years with a hospital admission in 2013, 2242 were hospitalised to geriatric wards, of which we included 715 in our analysis (see Fig. 1). The mean age of the study population was 82.5 years (SD = 7.8 range 65–101), and 64.8% were female. The mean length of hospital stay was 5.8 days (SD = 3.8 range 1–32). The most common medical diagnosis (identified from reimbursement codes) were hypertension (56.8%), atherosclerotic and cardiovascular disease (34.3%), mood disorders (19.3%), heart failure (17.9%), gastro-oesophageal reflux disease (17.9%), atrial fibrillation (14.1%) and chronic pain (13.8%).

### Medication and PIM use

After hospitalisation, the mean number of medications increased from 6.5 (SD = 3.5) per patient to 7.5 (SD = 3.5) (CI:1.2–0.8 *p* < 0.001), with a similar increase across all age groups. The medications prescribed to more patients after hospitalisation were paracetamol, atorvastatin, calcium and vitamin D, pantoprazole, metoprolol and dipyridamole, while the combination of paracetamol and codeine and ethylmorphine were prescribed to fewer patients after hospitalisation.

According to the EU(7)-PIM list, the proportion of patients with PIMs increased from 62.4% before hospitalisation to 69.2% after hospitalisation (*p* < 0.001), see Table 1. The median number of PIMs per patient after hospitalisation was higher than before hospitalisation (*p* < 0.001). Most of the PIMs originated from medications belonging to ATC group N05, zopiclone being responsible for most PIMs. The PIMs mostly added after hospitalisation were dipyridamole, rivaroxaban, zopiclone and nifedipine, see Table 2. All PIMs identified by EU(7)-PIM are found in supplement 2.

**Table 1:** Number of PIMs identified per patient (*n* = 715) before and after hospitalisation to a geriatric ward

Number of PIMs	EU(7)-PIM				NORGEP-NH			
	PIMs before		PIMs after		PIMs before		PIMs after	
	n	%	n	%	n	%	n	%
**1**	227	31.7	249	34.8	129	18.0	130	18.2
**2**	142	19.9	148	20.7	108	15.1	117	16.4
**3**	45	6.3	70	9.8	73	10.2	73	10.2
**4**	22	3.1	20	2.8	28	3.9	27	3.8
**5**	7	1.0	7	1.0	10	1.4	12	1.7
**6**	2	0.3	–	–	5	0.7	3	0.4
**7**	1	0.1	–	–	3	0.4	–	–
**8**	–	–	1	0.1	–	–	–	–
**9**	–	–	–	–	1	0.1	–	–
**Patients with PIMs**	**446**	**62.4**	**495**	**69.2**	**357**	**49.9**	**362**	**50.6**

**Table 2:** Patients (*n* = 715) with PIMs identified with the EU(7)-PIM list before and after hospitalisation grouped at ATC-level 3 and with the most frequently prescribed medications highlighted

	Patients with PIMs									
	Before		After		Removed		Not changed		Added	
	n	%	n	%	n	%	n	%	n	%
**N05 Psycholeptics**	260	36.4	293	41.0	35	4.9	225	31.5	68	9.5
Zopiclone *(Dosage > 3.75 mg/day*)	190	26.6	208	29.1	31	4.3	159	22.2	49	6.9
Diazepam	56	7.8	50	7.0	27	3.8	29	4.1	21	2.9
Nitrazepam	26	3.6	21	2.9	8	1.1	18	2.5	3	0.4
Zolpidem	20	2.8	22	3.1	6	0.8	14	2.0	8	1.1
**C08 Calcium channel blockers**	45	6.3	49	6.9	14	2.0	31	4.3	18	2.5
Nifedipine	23	3.2	33	4.6	5	0.7	18	2.5	15	2.1
**N06 Psychoanaleptics**	42	5.9	36	5.0	14	2.0	28	3.9	8	1.1
Amitriptyline	18	2.5	14	2.0	7	1.0	11	1.5	3	0.4
**B01 Antithrombotic agents**	39	5.5	110	15.4	12	1.7	27	3.8	83	11.6
Dipyridamole	23	3.2	55	7.7	9	1.3	14	2.0	41	5.7
Dabigatran	10	1.4	17	2.4	3	0.4	7	1.0	10	1.4
Rivaroxaban	6	0.8	33	4.6	2	0.3	4	0.6	29	4.1
**N02 Analgesics**	37	5.2	48	6.7	21	2.9	16	2.2	32	4.5
Tramadol	6	0.8	33	4.6	2	0.3	4	0.6	29	4.1
**A10 Drugs used in diabetes**	31	4.3	31	4.3	5	0.7	26	3.6	5	0.7
Glimepiride	25	3.5	22	3.1	4	0.6	21	2.9	1	0.1
**G04 Urologicals**	35	4.9	32	4.5	13	1.8	22	3.1	10	1.4
**R05 Cough and cold preparations**	28	3.9	17	2.4	23	3.2	5	0.7	12	1.7
Ethylmorphine	28	3.9	17	2.4	23	3.2	5	0.7	12	1.7
**C01 Cardiac therapy**	23	3.2	25	3.5	5	0.7	18	2.5	7	1.0
Digoxin	15	2.1	19	2.7	4	0.6	11	1.5	8	1,1
**M01 Antiinflammatory and antirheumatic products**	22	3,1	15	2.1	17	2.4	5	0.7	10	1.4
**A03 Drugs for functional gastrointestinal disorders**	21	2.9	22	3.1	16	2.2	5	0.7	17	2.4
Metoclopramide	21	2.9	22	3.1	16	2.2	5	0.7	17	2.4
**R06 Antihistamines for systemic use**	16	2.2	14	2.0	6	0.8	10	1.4	4	0.6
**A02 Drugs for acid-related disorders**	14	2.0	15	2.1	3	0.4	11	1.5	4	0.6
**G03 Sex hormones and modulators of the genital system**	14	2.0	15	2.1	3	0.4	11	1.5	4	0.6
**J01 Antibacterials for systemic use**	12	1.7	12	1.7	12	1.7	–	0.0	12	1.7
**N04 Anti-parkinson drugs**	12	1.7	11	1.5	2	0.3	10	1.4	1	0.1
**A06 Drugs for constipation**	9	1.3	21	2.9	6	0.8	3	0.4	18	2.5
**C02 Antihypertensives**	9	1.3	7	1.0	2	0.3	7	1.0	*–*	–
**C07 Beta-blocking agents**	9	1.3	6	0.8	5	0.7	4	0.6	2	0.3
**C03 Diuretics**	7	1.0	4	0.6	4	0.6	3	0.4	1	0.1
**N03 Antiepileptics**	7	1.0	11	1.5	1	0.1	6	0.8	5	0.7
**A07 Antidiarrheals, intestinal anti-inflammatory/ anti-infective agents**	4	0.6	11	1.5		0.0	4	0.6	7	1.0
**M03 Muscle relaxants**	4	0.6	3	0.4	1	0.1	3	0.4	*–*	–
**R01 Nasal preparations**	3	0.4	3	0.4	3	0.4		0.0	3	0.4
**A04 Antiemetics and antinauseants**	1	0.1	1	0.1	1	0.1		0.0	1	0.1
**M04 Antigout preparations**	1	0.1	2	0.3	*–*	–	1	0.1	1	0.1
**C04 Peripheral vasodilators**	0	0.0	1	0.1	–	–	–	–	1	0.1

According to the NORGEP-NH list, the proportion of patients with a PIM did not change from before to after hospitalisation (49.9 to 50.6%) (*p* = 0.73), see Table 1, nor did the median number of PIMs per patient (*p* = 0.79). Also here zopiclone was responsible for most PIM. Disregarding zopiclone, we identified PIM use in 39.2 and 37.6% of the patients before and after hospitalisation. Table 3 summarise PIMs identified by the NORGEP-NH list.

**Table 3:** Patients (*n* = 715) with PIMs identified with the NORGEP-NH list before and after hospitalisation

	Patients with PIMs									
	Before		After		Removed		Not changed		Added	
	n	%	n	%	n	%	n	%	n	%
Part A: Single substance criteria										
1.Combination analgesic codein/paracetamol	94	13.1	83	11.6	47	6.6	47	6.6	36	5.0
2. Tricyclic antidepressants (TCAs)	25	3.5	17	2.4	11	1.5	14	2.0	3	0.4
3. Non-steroid anti-inflammatory drugs (NSAIDs)	47	6.6	27	3.8	31	4.3	16	2.2	11	1.5
4. First-generation antihistamines	26	3.6	29	4.1	8	1.1	18	2.5	11	1.5
5. Diazepam	56	7.8	50	7.0	27	3.8	29	4.1	21	2.9
6. Oxazepam: Dosage > 30 mg/day	10	1.4	11	1.5	7	1.0	3	0.4	8	1.1
7. Zopiclone: Dosage > 5 mg/day	144	20.1	142	19.9	28	3.9	116	16.2	26	3.6
8. Nitrazepam	26	3.6	21	2.9	8	1.1	18	2.5	3	0.4
9. Flunitrazepam	1	0.1	–	–	1	0.1	–	–	–	–
10. Chlometiazole	2	0.3	9	1.3	1	0.1	1	0.1	8	1.1
11. Regular use of hypnotics^a^	196	27.4	206	28.8	28	3.9	168	23.5	38	5.3
**Total part A**	**316**	**44.2**	**322**	**45.0**	**60**	**8.4**	**256**	**35.8**	**66**	**9.2**
Part B: Combinations to avoid										
12. Warfarin + NSAIDs	2	0.3	–	–	2	0.2	–	–	–	–
13. Warfarin + SSRIs/SNRIs^b^	13	1.8	13	1.8	5	0.7	8	1.1	5	0.7
14. Warfarin+ ciprofloxacin/ofloxacin/erythromycin/clarithromycin	3	0.4	2	0.3	3	0.4	–	–	2	0.3
15. NSAIDs/coxibs^c^ + ACE-inhibitors/AT2-antagonists	16	2.2	13	1.8	11	1.5	5	0.7	8	1.1
16. NSAIDs/coxibs + diuretics	8	1.1	7	1.0	7	1.0	1	0.1	6	0.8
17. NSAIDs/coxibs + glucocorticoids	6	0.8	6	0.8	3	0.4	3	0.4	3	0.4
18. NSAIDs/coxibs + SSRI/SNRIs	7	1.0	4	0.6	7	1.0	–	–	4	0.6
19. ACE-inhibitors^d^/AT2-antagonists^e^ + potassium or potassium-sparing diuretics	19	2.7	23	3.2	9	1.3	10	1.4	13	1.8
20. Beta blocking agents + cardioselective calcium antagonists	2	0.3	2	0.3	1	0.1	1	0.1	1	0.1
21. Erythromycin/clarithromycin + statins	1	0.1	2	0.3	1	0.1	–	–	2	0.3
22.Bisphosponate + proton pump inhibitors	18	2.5	22	3.1	4	0.6	14	2.0	8	1.1
23. Concomitant use of 3 or more psychotropics	52	7.3	65	9.1	18	2.5	34	4.8	31	4.3
24. Tramadol + SSRIs	2	0.3	7	1.0	1	0.1	1	0.1	6	0.8
25. Metoprolol + paroxetine/fluoxetine/bupropion	1	0.1	2	0.3	–	–	1	0.1	1	0.1
26. Metformin + ACE-Inhibitors/AT2-antagonists + diuretics	9	1.3	6	0.8	5	0.7	4	0.6	2	0.3
**Total part B**	**129**	**18.0**	**139**	**19.4**	**49**	**6.9**	**80**	**11.2**	**59**	**8.3**
**Total PART A and B**	**357**	**49.9**	**362**	**50.6**	**73**	**10.2**	**284**	**39.7**	**78**	**10.9**

^a^ regular use defined as dispensing of 60 DDD or more in the 120-day period

^b^ selective serotonin reuptake inhibitors/selective norepinephrine reuptake inhibitors

^c^cyclooxygenase-2-selective inhibitors

^d^angiotensin-converting enzyme inhibitors

^e^ angiotensin II receptor antagonists

**Fig. 2:**
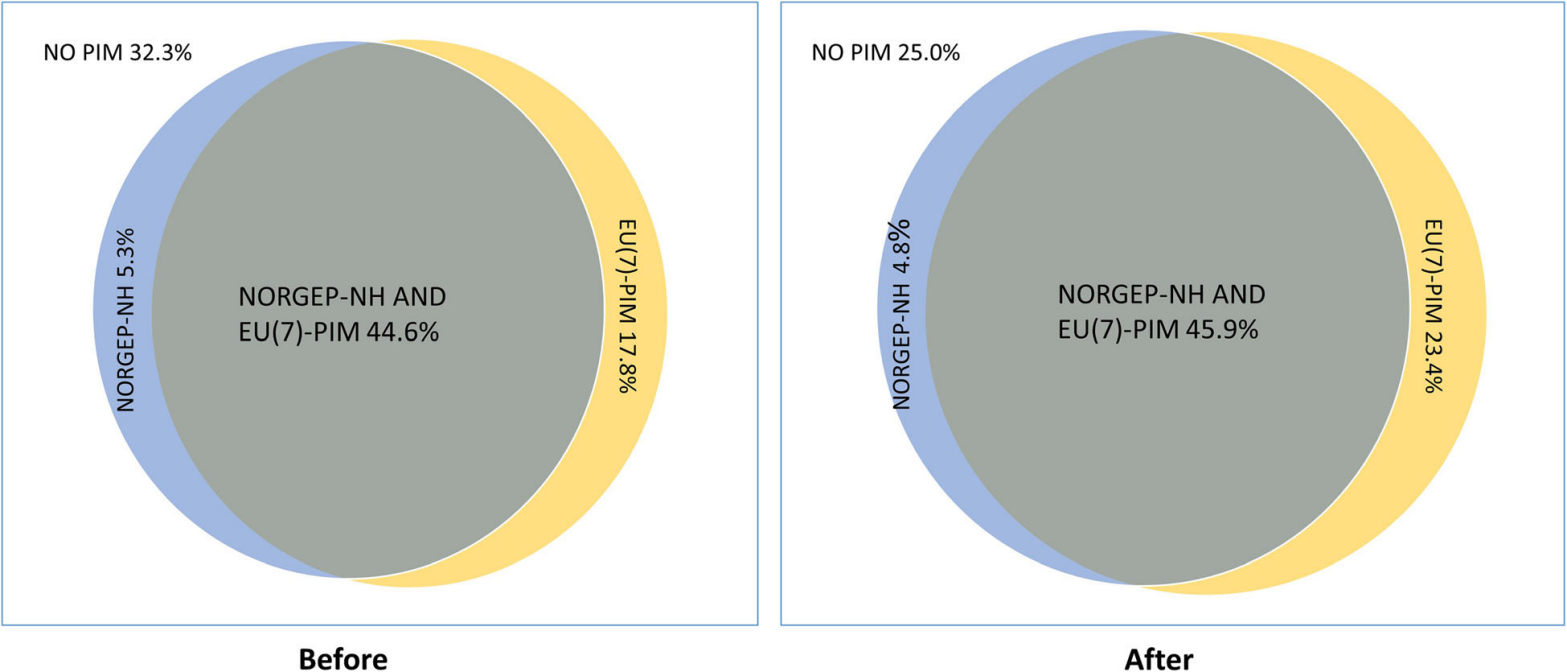
Proportion of study population identified as PIM users before and after hospitalisation with the EU(7)-PIM list and the NORGEP-NH list

Overall, we identified a higher prevalence of PIM users with the EU(7)-PIM list compared to the NORGEP-NH list. Before hospitalisation, the tools agreed on the classifications of patients as PIM users or non-PIM users in 76.9% of patients (44.6% PIM users in both tools) and 71.9% after hospitalisation (45.9% PIM users with both tools) see Fig. 2. If excluding zopiclone, responsible for most PIMs in both tools, the agreement between the tools decreased, to only 28% after hospitalisation.

## Discussion

In this study, we have shown the feasibility of applying health registry data for the identification of changes in PIM use in an older patient population admitted to hospitals in Norway. From the registry data, we were able to identify PIM use, compare PIM use before and after hospitalisation to a geriatric ward, and to compare the application of two different explicit PIM lists. Our study shows that the number of medications used increased significantly after hospitalisation to geriatric wards, which was also the case for PIM use according to the EU(7)-PIM list.

Applying registry data to investigate the effect of hospitalisation on PIM use is a novel approach. Although the registries did not contain information like a full list of medical diagnosis and laboratory data, we were able to apply most of the criteria and identify changes in PIMs. Previous studies have collected medication use data from hospital admission and discharge summaries [7–9]. Discharge summaries may not be fully representative for actual medication use after hospitalisation, as changes suggested by hospital physicians in discharge summaries are not necessarily effected in primary care [14]. There are numerous reasons for recommendations not being followed, but the most important may be poor communication between primary and secondary care [15]. The changes observed in medications use and PIMs after discharge in our study may be a result of prescriptions from both hospital and primary care physicians, as in real life.

### Increase in medication use and PIM use

There may be many reasons why medication and PIM use increased after hospitalisation, the most important perhaps being the nature of a hospitalisation, implying an acute illness or event where a need for new medications is expected [14, 16]. Most studies investigating the impact of hospitalisation on medication use have, similar to us, found an increase in the number of medications [8, 9, 14, 17]. If we assess the clinical impact of such an increase in an older population, it is not without risk. Polypharmacy has been associated with non-adherence to medication therapy, drug-interactions, ADEs, and readmissions [18, 19]. Increasing the number of medications prescribed also increases the risk of PIM-prescribing [20, 21]. Prescribing new medications to patients should prompt a medication review to optimize medication therapy.

We identified no reduction in PIM use, and this finding is coherent with results from studies investigating the impact of hospitalisation on PIM use in general. In a large longitudinal study from Ireland, using data from general practice records, hospital admissions were found to be independently associated with PIM-prescribing [22]. Norwegian studies examining the impact of hospitalisation on PIM use also support our findings. Bakken et al. found that stays in an intermediate-care nursing home unit or hospital wards increased PIM use identified by the NORGEP list from 24.1 to 34.8% of the population [23]. In two other Norwegian studies, no significant changes in PIM use were identified from admittance to discharge in geriatric and medical wards [24, 25]. International studies show conflicting results on the effect of a geriatric ward stay on PIMs [7–9].

### The type of PIMs identified

Although we found no overall reduction in PIM use, PIM changes occurred on the patient level. A large proportion of patients actually had PIMs removed, while an equal or larger proportion of patients had PIMs added (Tables 2 and 3). The most frequently identified PIMs with both tools were hypnotics, and zopiclone in particular. Nearly 30% of our study population used zopiclone ≥3.75 mg after hospitalisation (Table 2), a result supported by other Norwegian studies [26]. Given the considerable evidence relating hypnotics to ADEs in older adults, the widespread use of zopiclone is alarming, and interventions are warranted [27].

### Difference between PIM identification tools

This study suggests that the identification of PIMs is highly dependent on the tools applied, which was also the argument for applying two different PIM-lists. We found them to agree on the identification of PIM users in 76.9% before and 71.9% after hospitalisation. The EU(7)-PIM list, including 263 criteria is more sensitive but less specific than other tools, and thus identifies a higher prevalence of PIM use than the country-specific PIM lists [28]. In contrast the NORGEP-NH list only includes 34 criteria. We acknowledge that other criteria list also could have been used, however, to be applicable some of them require additional clinical information that is not recorded in our health registries, i.e. the Screening tool of older people’s prescriptions (STOPP) and screening tool to alert to right treatment (START) [29].

Looking into the specific difference between these two tools, the increase in PIMs identified by the EU(7)-PIM list after hospitalisation is primarily driven by the increased use of dipyridamole and direct oral anticoagulants (DOACs), which are not included in the NORGEP-NH list. A Norwegian geriatric hospital ward receives many stroke patients and increased use of antithrombotic agents is expected because extended-release dipyridamole in combination with aspirin is the first-line treatment for stroke according to Norwegian guidelines [30]. Consequently, an increase in dipyridamole use after a stay in a geriatric ward is regarded as appropriate in Norway. The EU(7)-PIM list also includes DOACs as inappropriate because of limited information on use in older adults and the risk of bleeding events [12]. This is not in accordance with one of the most popular and investigated PIM lists, i.e. the STOPP/START LIST [29], where failure to start DOACs in patients with chronic atrial fibrillation is defined as a potentially prescribing omission in the older adults [29]. There are obvious discrepancies between the different PIM identification lists concerning what is considered inappropriate prescribing. Consequently, we may not consider all PIMs identified by the EU(7)-PIM list to represent inappropriate prescribing in our population. Unlike the START/STOPP-list [6], the relationship between the EU(7)-PIM list and the NORGEP-NH list and adverse health outcomes in older adults is yet to be established. Research is needed to validate the ability of these newly developed PIM lists to identify patients at risk of ADEs. Applying explicit criteria PIM lists in direct patient care should always be done with individual clinical judgement.

Admittance to a geriatric ward is an opportunity to improve the quality of medication use in older patients. Geriatric wards, being tailored to care for older patients, should have the expertise to improve the appropriateness of medical treatment. Future research should find means to make a hospitalisation an opportunity for reducing PIMs in older patients. Pharmacist interventions have been shown to improve the appropriateness of prescribing at discharge [31], but in Norway, few geriatric wards had in 2013 included clinical pharmacists in their teams. Given the complexity of medication optimisation, a patient-focused multidisciplinary intervention targeting both primary and secondary care should be developed.

### Strengths and limitations

To our knowledge, our study is the first to use health registry data to investigate the impact of a geriatric ward stay on medication and PIM use on a national level. It is also the first study to apply the EU(7)-PIM list to a Norwegian population and to compare it to the country-specific NORGEP-NH list [29]. The main strength of our study is the quality of our health registry data enabling identification of all patients admitted to geriatric hospital wards and all prescription medications dispensed to community-dwelling patients.

The main limitation of this study is our definition of medication use as “all medications dispensed from the pharmacy during 120 days before or after hospitalisation”. This will likely overestimate use as patients may not use all of the medicines dispensed. On the other hand, compared to previous studies investigating the impact of geriatric ward stays on PIM use, we know for certain that the medications have been dispensed from the pharmacy, both before and after hospitalisation. A second limitation is that we could not apply all of the criteria in the EU(7)-PIM list because of limitations in our dataset. For example, use of proton pump inhibitors (PPI) for more than 8 weeks were excluded from our analysis, but is found to be the most frequent PIM identified with the EU(7)-PIM list [28]. A third limitation is that the provision of geriatric services and the criteria for admission to geriatric wards may be different in-between countries, and our results may not be directly transferable to other healthcare systems. A fourth limitation is that we excluded 1527 of the 2242 patients who had a hospital stay in a geriatric ward in 2013, mostly because of hospitalisations or lack of prescriptions in 120 days surrounding the index stay (Fig. 1). The population we have selected may be healthier than the average patients at geriatric wards because they only had one hospitalisation in 240 days and because lack of prescriptions in this population often means that they reside in a nursing home. This may introduce selection bias into our study, and limit the generalisability of our finding to the average patients at geriatric wards.

## Conclusion

Applying health registry data for identification of change in medication and PIM use after hospitalisation to geriatric wards in Norway is feasible. Medication use seems to increase significantly after hospitalisation to a geriatric ward. PIM use is prevalent both before and after hospitalisation, and did not identify any reduction after hospitalisation. A subset of the EU(7)-PIM and the NORGEP-NH list part A and B have a more than 70% agreement on the classification of patients as PIM users, but do not agree on whether PIM use increases after hospitalisation. More research is needed to validate if the increase in PIM use seen after hospitalisation with the EU(7)-PIMs list truly represent a risk of ADEs.

## Supplementary information

**Additional file 1: Online Resource 2.** The table shows medications from the EU(7)-PIM-List [1] that are included in our analysis and the adjustments that are done. For some of the medications, we include only some package sizes or strengths, while others we had to be excluded due to limitations in our dataset. Many of the medications in the list are not licensed in Norway but are not excluded as some patients may be allowed to use special imported non licensed medication.

**Additional file 2: Online resource 3.** All PIMs identified with the EU (7)-PIM list by ATC-level 5.

## Data Availability

The datasets generated and/or analysed during the current study are not publicly available due to restrictions from the Norwegian data protection authority and risk of identifying patients when linking registers but are available from the corresponding author on reasonable request.

## Abbreviations

ADE: Adverse drug events
ATC-code: Anatomical Therapeutic Chemical Code
DDD: Defined daily doses
EU(7)-PIM: European Union (EU)(7)-PIM list
NORGEP-NH: Norwegian General Practice – Nursing Home
PIM: Potentially inappropriate medications
STOPP: Screening tool of older people’s prescriptions
START: Screening tool to alert to right treatment lists
