# Supplementary material for: The impact of hospitalisation to geriatric wards on the use of medications and potentially inappropriate medications - a health register study
Source: BMC Geriatr. 2020 Jun 1;20:190. doi: 10.1186/s12877-020-01585-w (PMC7268415; doi:10.1186/s12877-020-01585-w)
Supplement: Supplementary file 1 — Additional file 1: Online Resource 2. The table shows medications from the EU(7)-PIM-List [1] that are included in our analysis and the adjustments that are done. For some of the medications, we include only some package sizes or strengths, while others we had to be excluded due to limitations in our dataset. Many of the medications in the list are not licensed in Norway but are not excluded as some patients may be allowed to use special imported non licensed medication. [file 12877_2020_1585_MOESM1_ESM.docx]

**Online Resource 2**

**The table shows medications from the EU (7)-PIM-List [1] that are included in our analysis, and the adjustments done. For some of the medications we include only some package sizes or strengths, while others we had to be excluded do to limitations in our dataset. Many of the medications in the list are not licensed in Norway but are not excluded as some patients may be allowed to use special imported non licensed medication.**

| **ATC-Code** | **Potentially inappropriate** | **Included** | **Excluded** | **Included with modifications** | **Added** | **Comment** |
| --- | --- | --- | --- | --- | --- | --- |
| A02AA04 | Magnesium hydroxide | X |  |  |  |  |
| A02AB, A02AD | Aluminium-containing antacids | X |  |  |  | Medication group |
| A02AD01 | Ordinary salt combinations |  |  |  | X | Group specified, added ATC level 5 for medications in group available In Norway. |
| A02BA01 | Cimetidine | X |  |  |  |  |
| A02BA02 | Ranitidine | X |  |  |  |  |
| A02BA03 | Famotidine | X |  |  |  |  |
| A02BC | Proton pump inhibitors (PPI) (>8 weeks) e.g. omeprazole, pantoprazole |  | X |  |  | Excluded because our data cannot separate use over or under 8 weeks |
| A03AA04 | Mebeverine^c^ | X |  |  |  |  |
| A03AA05 | Trimebutine | X |  |  |  |  |
| A03AA08 | Dihexyverine | X |  |  |  |  |
| A03AB06 | Otilonium bromide | X |  |  |  |  |
| A03AB17 | Tiemonium (iodide) | X |  |  |  |  |
| A03AX04 | Pinaverium^c^ | X |  |  |  |  |
| A03BA03 | Hyoscyamine | X |  |  |  |  |
| A03BA04 | Belladonna alkaloids | X |  |  |  |  |
| A03CA02 | Clidinium-Chlordiazepoxide | X |  |  |  |  |
| A03DA02 | Pitofenone | X |  |  |  |  |
| A03FA01 | Metoclopramide | X |  |  |  |  |
| A03FA03 | Domperidone (>30 mg/d)^c^ |  | X |  |  | Excluded, not licensed in Norway and no data on dose in our dataset |
| A03FA05 | Alizapride | X |  |  |  |  |
| A04AB02 | Dimenhydrinate | X |  |  |  |  |
| A04AD01 | Scopolamine | X |  |  |  |  |
| A04AD05 | Metopimazine | X |  |  |  |  |
| A06AA01 | Viscous paraffin (=Liquid paraffin) | X |  |  |  |  |
| A06AA02 | Docusate sodium (oral) |  |  | X |  | Removed suppository as criteria specified oral use |
| A06AB02 | Bisacodyl (>3 days) |  |  | X |  | Included package size of 100 and 250 tablets as these are intended for prolonged use. |
| A06AB05 | Castor oil (=Ricinus communis, =Neoloid) | X |  |  |  |  |
| A06AB06 | Senna glycosides | X |  |  |  |  |
| A06AB07 | Cascara sagrada | X |  |  |  |  |
| A06AB08 | Sodium picosulfate | X |  |  |  |  |
| A06AB13 | Aloe | X |  |  |  |  |
| A06AX05 | Prucalopride | X |  |  |  |  |
| A07DA01 | Diphenoxylate-Atropine | X |  |  |  |  |
| A07DA03 | Loperamide (>2 days) |  |  | X |  | Included package size of 100 and 250 tablets as these are intended for prolonged use. |
| A07XA04 | Racecadotril | X |  |  |  |  |
| no ATC, treatment concept PIM | Insulin, sliding scale |  | X |  |  | No information in dataset if insulin is used in sliding scale |
| A10BB01 | Glibenclamide | X |  |  |  |  |
| A10BB02 | Chlorpropamide | X |  |  |  |  |
| A10BB06 | Carbutamide | X |  |  |  |  |
| A10BB07 | Glipizide | X |  |  |  |  |
| A10BB12 | Glimepiride | X |  |  |  |  |
| A10BF01 | Acarbose | X |  |  |  |  |
| A10BG03 | Pioglitazone | X |  |  |  |  |
| A10BH01 | Sitagliptine | X |  |  |  |  |
| A10BH02 | Vildagliptine | X |  |  |  |  |
| B01AA07 | Acenocoumarol | X |  |  |  |  |
| B01AC05 | Ticlopidine | X |  |  |  |  |
| B01AC07 | Dipyridamole | X |  |  |  |  |
| B01AC22 | Prasugrel | X |  |  |  |  |
| B01AE07 | Dabigatran^c^ | X |  |  |  |  |
| B01AF01 | Rivaroxaban^c^ | X |  |  |  |  |
| B01AF02 | Apixaban^c^ | X |  |  |  |  |
| B03AA | Iron supplements / Ferrous sulfate (>325 mg/d) |  | X |  |  | Largest tablets strength is 200 mg no information on number of tablets prescribed each day. |
| C01AA02 | Acetyldigoxin | X |  |  |  |  |
| C01AA04 | Digitoxin | X |  |  |  |  |
| C01AA05 | Digoxin | X |  |  |  |  |
| C01AA08 | Metildigoxin | X |  |  |  |  |
| C01BA01 | Quinidine | X |  |  |  |  |
| C01BA02 | Procainamide | X |  |  |  |  |
| C01BA03 | Disopyramide | X |  |  |  |  |
| C01BA51 | Quinidine in combination with verapamil | X |  |  |  |  |
| C01BC03 | Propafenone | X |  |  |  |  |
| C01BC04 | Flecainide | X |  |  |  |  |
| C01BD01 | Amiodarone | X |  |  |  |  |
| C01BD07 | Dronedarone | X |  |  |  |  |
| C01EB15 | Trimetazidine | X |  |  |  |  |
| C01EB17 | Ivabradine | X |  |  |  |  |
| C02AA02 | Reserpine | X |  |  |  |  |
| C02AB01 | Methyldopa | X |  |  |  |  |
| C02AC01 | Clonidine | X |  |  |  |  |
| C02AC02 | Guanfacine | X |  |  |  |  |
| C02AC05 | Moxonidine | X |  |  |  |  |
| C02AC06 | Rilmenidine | X |  |  |  |  |
| C02CA01 | Prazosin | X |  |  |  |  |
| C02CA04 | Doxazosin | X |  |  |  |  |
| C02CA06 | Urapidil | X |  |  |  |  |
| C02CC02 | Guanethidine | X |  |  |  |  |
| C02DB02 | Hydralazine | X |  |  |  |  |
| C03DA01 | Spironolactone (>25 mg/d)^c^ | X |  | X |  | Included dispensed tablets with a strength over 25 mg |
| C04AD03 | Pentoxifylline | X |  |  |  |  |
| C04AE02 | Nicergoline | X |  |  |  |  |
| C04AE04 | Dihydroergocristine | X |  |  |  |  |
| C04AE54 | Raubasine-Dihydroergocristine | X |  |  |  |  |
| C04AX01 | Cyclandelate (=Cyclospasmol) | X |  |  |  |  |
| C04AX07 | Vincamine | X |  |  |  |  |
| C04AX10 | Moxisylyte | X |  |  |  |  |
| C04AX17 | Vinburnine | X |  |  |  |  |
| C04AX20 | Buflomedil | X |  |  |  |  |
| C04AX21 | Naftidrofuryl | X |  |  |  |  |
| C05CA05 | Hidrosmin | X |  |  |  |  |
| C05CA07 | Escin (=Aescin) | X |  |  |  |  |
| C05CA51 | Vincamine-Rutoside | X |  |  |  |  |
| C05CA54 | Troxerutin-Vincamine | X |  |  |  |  |
| C07AA02 | Oxprenolol | X |  |  |  |  |
| C07AA03 | Pindolol | X |  |  |  |  |
| C07AA05 | Propranolol | X |  |  |  |  |
| C07AA07 | Sotalol | X |  |  |  |  |
| C07AA12 | Nadolol | X |  |  |  |  |
| C07AG01 | Labetalol | X |  |  |  |  |
| C08CA04 | Nicardipine | X |  |  |  |  |
| C08CA05 | Nifedipine (non-sustained-release) | X |  |  |  |  |
| C08CA05 | Nifedipine (sustained-release) | X |  |  |  |  |
| C08DA01 | Verapamil | X |  |  |  |  |
| C08DB01 | Diltiazem | X |  |  |  |  |
| C10AD02 | Niacin (=Nicotinic acid) | X |  |  |  |  |
| G03C | Oestrogen (oral) | X |  |  |  | Medication group |
| G03CA03 | Østradiol |  |  | X | X | Included only tablets, removed vaginal tablets |
| G03CA04 | Østriol |  |  | X | X | Included only tablets, removed vaginal tablets |
| G03CX01 | Tibolon |  |  |  | X |  |
| G04BD02 | Flavoxat | X |  |  |  |  |
| G04BD04 | Oxybutynine (non-sustained-release) | X |  |  |  |  |
| G04BD04 | Oxybutynine (sustained-release) | X |  |  |  |  |
| G04BD07 | Tolterodine (non-sustained-release) | X |  |  |  |  |
| G04BD07 | Tolterodine (sustained-release) | X |  |  |  |  |
| G04BD08 | Solifenacin | X |  |  |  |  |
| G04BD09 | Trospium | X |  |  |  |  |
| G04BD10 | Darifenacin | X |  |  |  |  |
| G04BD11 | Fesoterodin | X |  |  |  |  |
| G04CA03 | Terazosin | X |  |  |  |  |
| J01MA01 | Ofloxacin | X |  |  |  |  |
| J01XE01 | Nitrofurantoin (>1 week) |  |  | X |  | Included only 100 package of 50 mg as these are intended for prolonged use |
| M01AA01 | Phenylbutazone | X |  |  |  |  |
| M01AB01 | Indometacin | X |  |  |  |  |
| M01AB05 | Diclofenac | X |  |  |  |  |
| M01AB11 | Acemetacin | X |  |  |  |  |
| M01AB15 | Ketorolac | X |  |  |  |  |
| M01AB16 | Aceclofenac | X |  |  |  |  |
| M01AC01 | Piroxicam | X |  |  |  |  |
| M01AC05 | Lornoxicam | X |  |  |  |  |
| M01AC06 | Meloxicam | X |  |  |  |  |
| M01AE01 | Ibuprofen (>3 x 400 mg/d or for a period longer than one week)^c^ |  | X |  |  | No information on dose and length of therapy in dataset. |
| M01AE02 | Naproxen (>2 x 250 mg/d or for a period longer than one week)^c^ |  | X |  |  | No information on dose and length of therapy in dataset. |
| M01AE03 | Ketoprofen | X |  |  |  |  |
| M01AE09 | Flurbiprofen | X |  |  |  |  |
| M01AE17 | Dexketoprofen | X |  |  |  |  |
| M01AG01 | Mefenamic acid | X |  |  |  |  |
| M01AH01 | Celecoxib | X |  |  |  |  |
| M01AH05 | Etoricoxib | X |  |  |  |  |
| M01AX01 | Nabumetone | X |  |  |  |  |
| M03BA02 | Carisoprodol | X |  |  |  |  |
| M03BA03 | Methocarbamol | X |  |  |  |  |
| M03BC01 | Orphenadrine | X |  |  |  |  |
| M03BX01 | Baclofen | X |  |  |  |  |
| M03BX02 | Tizanidine | X |  |  |  |  |
| M03BX07 | Tetrazepam | X |  |  |  |  |
| M03BX08 | Cyclobenzaprine | X |  |  |  |  |
| M04AC01 | Colchicin | X |  |  |  |  |
| M05BX03 | Strontium ranelate | X |  |  |  |  |
| M09AA | Quinine and derivatives |  | X |  |  | Medication group. No medications in this group is licensed for use in Norway |
| N02AB02 | Pethidine (=Meperidine) | X |  |  |  |  |
| N02AD01 | Pentazocine | X |  |  |  |  |
| N02AX02 | Tramadol (sustained-release) | X |  |  |  |  |
| N02AX02 | Tramadol (non-sustained-release) | X |  |  |  |  |
| N07BC02 | Methadone | X |  |  |  |  |
| N02BA01 | Acetylsalicylic acid (>325 mg) |  | X |  |  | No information on dose over 160 mg, larger strengths not available on prescription in Norway |
| N02CA02 | Ergotamine | X |  |  |  |  |
| N02CC | Triptanes (e.g. Sumatriptan, Eletriptan, Naratriptan, Zolmitriptan) | X |  |  |  | Medication group. Added medications on the Norwegian market. |
| N02CC01 | Sumatriptan |  |  |  | X |  |
| N02CC02 | Naratriptan |  |  |  | X |  |
| N02CC03 | Zolmitriptan |  |  |  | X |  |
| N02CC04 | Rizatriptan |  |  |  | X |  |
| N02CC05 | Almotriptan |  |  |  | X |  |
| N02CC06 | Eletriptan |  |  |  | X |  |
| N02CC07 | Frovatriptan |  |  |  | X |  |
| N03AA02 | Phenobarbital | X |  |  |  |  |
| N03AB02 | Phenytoin | X |  |  |  |  |
| N03AE01 | Clonazepam | X |  |  |  |  |
| N03AF01 | Carbamazepine | X |  |  |  |  |
| N03AX11 | Topiramate | X |  |  |  |  |
| N04AA01 | Trihexyphenidyl | X |  |  |  |  |
| N04AA02 | Biperiden | X |  |  |  |  |
| N04AA12 | Tropatepin | X |  |  |  |  |
| N04AC01 | Benzatropine | X |  |  |  |  |
| N04BB01 | Amantadine | X |  |  |  |  |
| N04BC01 | Bromocriptine | X |  |  |  |  |
| N04BC02 | Pergolide | X |  |  |  |  |
| N04BC03 | Dihydroergocryptine | X |  |  |  |  |
| N04BC04 | Ropinirole^c^ | X |  |  |  |  |
| N04BC05 | Pramipexole^c^ | X |  |  |  |  |
| N04BC06 | Cabergoline^c^ | X |  |  |  |  |
| N04BC08 | Piribedil | X |  |  |  |  |
| N04BC09 | Rotigotine | X |  |  |  |  |
| N04BD01 | Selegiline | X |  |  |  |  |
| N05AA01 | Chlorpromazine | X |  |  |  |  |
| N05AA02 | Levomepromazine | X |  |  |  |  |
| N05AA04 | Acepromazine | X |  |  |  | Two ATC-codes in one criteria. |
| N05BA05 | Clorazepate | X |  |  |  |  |
| N05AA06 | Cyamemazine | X |  |  |  |  |
| N05AB02 | Fluphenazine | X |  |  |  |  |
| N05AB03 | Perphenazine | X |  |  |  |  |
| N05AB04 | Prochlorperazine | X |  |  |  |  |
| N05AB06 | Trifluoperazine | X |  |  |  |  |
| N05AC01 | Propericiazine (=Periciazine) | X |  |  |  |  |
| N05AC02 | Thioridazine | X |  |  |  |  |
| N05AC04 | Pipotiazine | X |  |  |  |  |
| N05AD01 | Haloperidol (>2 mg single dose; >5mg/d) |  | X |  |  | No data on use of doses over 1 mg per day (larges tablet strength) |
| N05AD08 | Droperidol | X |  |  |  |  |
| N05AE03 | Sertindole | X |  |  |  |  |
| N05AE04 | Ziprasidone | X |  |  |  |  |
| N05AF01 | Flupentixole | X |  |  |  |  |
| N05AF03 | Chlorprothixen | X |  |  |  |  |
| N05AF05 | Zuclopenthixol | X |  |  |  |  |
| N05AG02 | Pimozide | X |  |  |  |  |
| N05AH02 | Clozapine | X |  |  |  |  |
| N05AH03 | Olanzapine (>10 mg/d) |  |  | X |  | Include only tablets with strength over 10 mg |
| N05AN01 | Lithium | X |  |  |  |  |
| N05AX08 | Risperidone (>6 weeks) |  | X |  |  | Excluded because our data cannot separate use over or under 6 weeks |
| N05AX12 | Aripiprazole | X |  |  |  |  |
| N05BA01 | Diazepam | X |  |  |  |  |
| N05BA02 | Chlordiazepoxide | X |  |  |  |  |
| N05BA03 | Medazepam | X |  |  |  |  |
| N05BA04 | Oxazepam (>60 mg/d) |  | X |  |  | No data on use of doses over 30 mg per day (larges tablet strength available) |
| N05BA05 | Dipotassium clorazepate | X |  |  |  | ATC-code included already |
| N05BA06 | Lorazepam (>1 mg/d) |  | X |  |  | Not available in Norway, not able to adjust for dosing |
| N05BA08 | Bromazepam | X |  |  |  |  |
| N05BA09 | Clobazam | X |  |  |  |  |
| N05BA11 | Prazepam | X |  |  |  |  |
| N05BA12 | Alprazolam | X |  |  |  |  |
| N05BA13 | Halazepam | X |  |  |  |  |
| N05BA16 | Nordazepam | X |  |  |  |  |
| N05BA18 | (Ethyl-) Loflazepate | X |  |  |  |  |
| N05BA21 | Clotiazepam (>5 mg/d) |  | X |  |  | Not available in Norway, not able to adjust for dosing |
| N05BC01 | Meprobamate | X |  |  |  |  |
| N05CC01 | Chloralhydrate | X |  |  |  |  |
| N05CD01 | Flurazepam | X |  |  |  |  |
| N05CD02 | Nitrazepam | X |  |  |  |  |
| N05CD03 | Flunitrazepam | X |  |  |  |  |
| N05CD04 | Estazolam | X |  |  |  |  |
| N05CD05 | Triazolam | X |  |  |  |  |
| N05CD06 | Lormetazepam (>0.5 mg/d) |  | X |  |  | Not available in Norway, not able to adjust for dosing |
| N05CD07 | Temazepam | X |  |  |  |  |
| N05CD08 | Midazolam | X |  |  |  |  |
| N05CD09 | Brotizolam (>0.125 mg/d) |  | X |  |  | Not available in Norway, not able to adjust for dosing |
| N05CD10 | Quazepam | X |  |  |  |  |
| N05CD11 | Loprazolam (>0.5 mg/d)^c^ |  | X |  |  |  |
| N05CF01 | Zopiclone (>3.75 mg/d) |  |  | X |  | Included only dispensing of 5 mg and 7.5 mg tablets |
| N05CF02 | Zolpidem (>5 mg/d) |  |  | X |  | Included only dispensing of 10 mg tablets |
| N05CF03 | Zaleplone (>5 mg/d) |  | X |  |  |  |
| N05CM02 | Clomethiazole | X |  |  |  |  |
| N05CM06 | Propiomazine | X |  |  |  |  |
| No ATC | Aceprometazine |  | X |  |  | Excluded, no ATC |
| N06AA01 | Desipramine | X |  |  |  |  |
| N06AA02 | Imipramine | X |  |  |  |  |
| N06AA04 | Clomipramine | X |  |  |  |  |
| N06AA06 | Trimipramine | X |  |  |  |  |
| N06AA09 | Amitriptyline | X |  |  |  |  |
| N06AA10 | Nortriptyline | X |  |  |  |  |
| N06AA12 | Doxepin | X |  |  |  |  |
| N06AA16 | Dosulepin | X |  |  |  |  |
| N06AA17 | Amoxapine | X |  |  |  |  |
| N06AA21 | Maprotiline | X |  |  |  |  |
| N06AB03 | Fluoxetine | X |  |  |  |  |
| N06AB05 | Paroxetine | X |  |  |  |  |
| N06AB08 | Fluvoxamine | X |  |  |  |  |
| N06AF04 | Tranylcypromine | X |  |  |  |  |
| N06AX12 | Bupropion | X |  |  |  |  |
| N06AX16 | Venlafaxine | X |  |  |  |  |
| N06AX18 | Reboxetine | X |  |  |  |  |
| N06BA04 | Methylphenidat | X |  |  |  |  |
| N06BX03 | Piracetam | X |  |  |  |  |
| N06DX02 | Ginkgo biloba | X |  |  |  |  |
| C04AE01 | Ergoloid mesylate (dihydroergotoxine) | X |  |  |  |  |
| N07AB02 | Bethanechol | X |  |  |  |  |
| R01BA01 | Norephedrine (=Phenylpropanolamine) | X |  |  |  |  |
| R01BA02 | Pseudoephedrine | X |  |  |  |  |
| R03CC03 | Terbutaline (oral) |  |  | X |  | Removed dispensing of injections (same ATC-code) |
| R03DA04 | Theophylline | X |  |  |  |  |
| R05DA01 | Ethylmorphine | X |  |  |  |  |
| R05DA04 | Codeine (>2 weeks) |  | X |  |  | Excluded because our data cannot separate use over or under 2 weeks. |
| R05DA09 | Dextrometorphan | X |  |  |  |  |
| R06AA02 | Diphenhydramine | X |  |  |  |  |
| R06AA04 | Clemastine | X |  |  |  |  |
| R06AA08 | Carbinoxamine | X |  |  |  |  |
| R06AA09 | Doxylamine | X |  |  |  |  |
| R06AB01 | Brompheniramine | X |  |  |  |  |
| R06AB02 | Dexchlorpheniramine | X |  |  |  |  |
| R06AB03 | Dimetindene | X |  |  |  |  |
| R06AB04 | Chlorpheniramine (=Chlorphenamine) | X |  |  |  |  |
| R06AB05 | Pheniramine | X |  |  |  |  |
| R06AB52 | Dexchlorpheniramine-Betamethason | X |  |  |  |  |
| R06AC04 | Tripelennamine | X |  |  |  |  |
| R06AD01 | Alimemazine | X |  |  |  |  |
| R06AD02 | Promethazine | X |  |  |  |  |
| R06AD07 | Mequitazine | X |  |  |  |  |
| R06AD08 | Oxomemazine | X |  |  |  |  |
| R06AE01 | Buclizine | X |  |  |  |  |
| R06AE03 | Cyclizine | X |  |  |  |  |
| R06AE05 | Meclozine | X |  |  |  |  |
| R06AX02 | Cyproheptadine | X |  |  |  |  |
| R06AX07 | Triprolidine | X |  |  |  |  |
| R06AX12 | Terfenadine | X |  |  |  |  |
| R06AX22 | Ebastine | X |  |  |  |  |
| R06AX23 | Pimethixene | X |  |  |  |  |
| N05BB01 | Hydroxyzine | X |  |  |  |  |

1 Renom-Guiteras A, Meyer G, Thurmann PA (2015) The EU(7)-PIM list: a list of potentially inappropriate medications for older people consented by experts from seven European countries. European journal of clinical pharmacology 71 (7): 861-875 DOI 10.1007/s00228-015-1860-9
