# Supplementary material for: The impact of hospitalisation to geriatric wards on the use of medications and potentially inappropriate medications - a health register study
Source: BMC Geriatr. 2020 Jun 1;20:190. doi: 10.1186/s12877-020-01585-w (PMC7268415; doi:10.1186/s12877-020-01585-w)
Supplement: Supplementary file 2 — Additional file 2: Online resource 3. All PIMs identified with the EU (7)-PIM list by ATC-level 5. [file 12877_2020_1585_MOESM2_ESM.docx]

Online resource 3.

All PIMs identified with the EU (7)-PIM list by ATC-level 5

|  | Number of PIMs | | Number of PIMs removed, not changed or added | | |
| --- | --- | --- | --- | --- | --- |
| ATC-code | PIM before | PIM after | Removed | Not changed | Added |
| N05CF01 | 190 | 208 | 31 | 159 | 49 |
| N05BA01 | 56 | 50 | 27 | 29 | 21 |
| N02AX02 | 36 | 45 | 20 | 16 | 29 |
| R05DA01 | 28 | 17 | 23 | 5 | 12 |
| N05CD02 | 26 | 21 | 8 | 18 | 3 |
| A10BB12 | 25 | 22 | 4 | 21 | 1 |
| B01AC07 | 23 | 55 | 9 | 14 | 41 |
| C08CA05 | 23 | 33 | 5 | 18 | 15 |
| A03FA01 | 21 | 22 | 16 | 5 | 17 |
| N05CF02 | 20 | 22 | 6 | 14 | 8 |
| N06AA09 | 18 | 14 | 7 | 11 | 3 |
| C01AA05 | 15 | 19 | 4 | 11 | 8 |
| A02BA02 | 14 | 15 | 3 | 11 | 4 |
| C08DA01 | 14 | 11 | 5 | 9 | 2 |
| G04BD08 | 13 | 12 | 5 | 8 | 4 |
| M01AB05 | 12 | 7 | 9 | 3 | 4 |
| N05BB01 | 12 | 16 | 3 | 9 | 7 |
| G03CA04 | 11 | 12 | 3 | 8 | 4 |
| J01XE01 | 11 | 11 | 11 | 0 | 11 |
| N05AA02 | 11 | 6 | 6 | 5 | 1 |
| R06AD01 | 11 | 10 | 4 | 7 | 3 |
| B01AE07 | 10 | 17 | 3 | 7 | 10 |
| G04BD07 | 10 | 8 | 4 | 6 | 2 |
| C02CA04 | 8 | 6 | 2 | 6 | 0 |
| C08DB01 | 8 | 5 | 4 | 4 | 1 |
| G04BD11 | 8 | 8 | 4 | 4 | 4 |
| N06AX16 | 8 | 8 | 2 | 6 | 2 |
| A10BH01 | 7 | 5 | 3 | 4 | 1 |
| C03DA01 | 7 | 4 | 4 | 3 | 1 |
| A06AB08 | 6 | 15 | 5 | 1 | 14 |
| B01AF01 | 6 | 33 | 2 | 4 | 29 |
| C07AA07 | 6 | 2 | 4 | 2 | 0 |
| N04BC05 | 6 | 4 | 2 | 4 | 0 |
| N05AF03 | 6 | 4 | 3 | 3 | 1 |
| C01AA04 | 5 | 3 | 2 | 3 | 0 |
| M01AH05 | 5 | 6 | 4 | 1 | 5 |
| N06AB05 | 5 | 7 | 1 | 4 | 3 |
| N06AA06 | 5 | 2 | 3 | 2 | 0 |
| A07DA03 | 4 | 11 | 0 | 4 | 7 |
| G04BD10 | 4 | 4 | 1 | 3 | 1 |
| N04BC04 | 4 | 4 | 0 | 4 | 0 |
| R06AB02 | 4 | 3 | 2 | 2 | 1 |
| A10BB07 | 3 | 4 | 0 | 3 | 1 |
| C01BC04 | 3 | 3 | 0 | 3 | 0 |
| C07AA05 | 3 | 4 | 2 | 1 | 3 |
| G03CA03 | 3 | 3 | 0 | 3 | 0 |
| M01AE03 | 3 | 0 | 3 | 0 | 0 |
| M03BX01 | 3 | 3 | 0 | 3 | 0 |
| N03AF01 | 3 | 6 | 0 | 3 | 3 |
| N06AB03 | 3 | 3 | 0 | 3 | 0 |
| R01BA01 | 3 | 3 | 3 | 0 | 3 |
| A06AB02 | 2 | 5 | 1 | 1 | 4 |
| M01AC01 | 2 | 0 | 2 | 0 | 0 |
| N02CC01 | 2 | 1 | 2 | 0 | 1 |
| N03AA02 | 2 | 1 | 1 | 1 | 0 |
| N05AB04 | 2 | 3 | 1 | 1 | 2 |
| N05AF01 | 2 | 2 | 0 | 2 | 0 |
| N05AN01 | 2 | 2 | 0 | 2 | 0 |
| N05CM02 | 2 | 9 | 1 | 1 | 8 |
| N06AX12 | 2 | 1 | 1 | 1 | 0 |
| A04AD01 | 1 | 1 | 1 | 0 | 1 |
| A06AB06 | 1 | 2 | 0 | 1 | 1 |
| A10BG03 | 1 | 1 | 0 | 1 | 0 |
| A10BH02 | 1 | 3 | 0 | 1 | 2 |
| C01BD01 | 1 | 1 | 0 | 1 | 0 |
| C02AC05 | 1 | 1 | 0 | 1 | 0 |
| J01MA01 | 1 | 1 | 1 | 0 | 1 |
| M03BA02 | 1 | 0 | 1 | 0 | 0 |
| M04AC01 | 1 | 2 | 0 | 1 | 1 |
| N03AB02 | 1 | 1 | 0 | 1 | 0 |
| N03AE01 | 1 | 3 | 0 | 1 | 2 |
| N04BC06 | 1 | 1 | 0 | 1 | 0 |
| N04AA02 | 1 | 1 | 0 | 1 | 0 |
| N05AB03 | 1 | 1 | 0 | 1 | 0 |
| N05AF05 | 1 | 1 | 0 | 1 | 0 |
| N05BA12 | 1 | 1 | 0 | 1 | 0 |
| N05CD03 | 1 | 0 | 1 | 0 | 0 |
| N06AB08 | 1 | 1 | 0 | 1 | 0 |
| N06AA10 | 1 | 1 | 0 | 1 | 0 |
| N06AA12 | 1 | 0 | 1 | 0 | 0 |
| R06AX22 | 1 | 1 | 0 | 1 | 0 |
| B01AF02 | 0 | 6 | 0 | 0 | 6 |
| C04AD03 | 0 | 1 | 0 | 0 | 1 |
| M01AC06 | 0 | 1 | 0 | 0 | 1 |
| M01AH01 | 0 | 1 | 0 | 0 | 1 |
| N02CC04 | 0 | 1 | 0 | 0 | 1 |
| N02CC06 | 0 | 1 | 0 | 0 | 1 |
| N04BD01 | 0 | 1 | 0 | 0 | 1 |
| N05CD08 | 0 | 2 | 0 | 0 | 2 |
| **SUM** | **788** | **878** | **286** | **502** | **376** |
